# Serum Biomarkers in Bullous Pemphigoid: A Systematic Review

**DOI:** 10.1177/12034754241266171

**Published:** 2024-07-29

**Authors:** Ryan S. Q. Geng, Elizabeth Wei, Bethany Wilken, Ronald G. Sibbald, Cathryn Sibbald

**Affiliations:** 1 Temerty School of Medicine, University of Toronto, Toronto, ON, Canada; 2 Faculty of Medicine, Queen’s University, Kingston, ON, Canada; 3 Dalla Lana School of Public Health & Division of Dermatology, Department of Medicine, University of Toronto, Toronto, ON, Canada; 4 Division of Pediatric Dermatology, The Hospital for Sick Children, University of Toronto, Toronto, ON, Canada

**Keywords:** bullous pemphigoid, biomarkers, coagulation cascade, immune pathways

## Abstract

**Introduction:**

Bullous pemphigoid (BP) is the most common type of subepidermal blistering disease, usually observed in the elderly population, with a mean age of presentation between 66 and 83 years. BP is a psychosocially ladened disease, with many patients experiencing negative body image, social isolation, and depression. The identification and validation of biomarkers in BP may further the understanding of disease pathogenesis, provide objective measures in assessing efficacy in clinical trials, and identify new targets for targeted therapy.

**Methods/Results:**

Two databases (Medline and Embase) were searched from database inception to September 2023. All published articles reporting on biomarker levels of BP patients in serum compared to healthy controls were included. A total of 877 unique articles were identified, resulting in the inclusion of 62 case-control studies reporting on a total of 1837 patients and 140 unique biomarkers. Biomarkers were categorized into T-cell mediated, B-cell mediated, innate immune system, and coagulation cascade pathway. The most notable biomarkers identified include increases in anti-BP180/230 immunoglobulin (Ig)G/E, total IgE, TNF-α, B-cell activating factor, interleukin-31, eosinophil cationic protein, MMP-9, and coagulation cascade biomarker levels. The results of this review provide the greatest support for a role of anti-BP180/230 autoantibodies, T_h_2 cells, eosinophils, and the coagulation cascade in the pathogenesis of BP.

**Conclusions:**

The pathogenesis of BP has an underlying autoimmune etiology centred around the production of autoantibodies against BP180/230, but increased T_h_2, eosinophil and coagulation cascade activity may be contributory.

## Introduction

Bullous pemphigoid (BP) is the most common type of subepidermal blistering disease. The mean age of presentation is between 66 and 83 years, with incidence rates between 2.4 and 21.7 new cases per million population. While BP is most prevalent in the elderly population, it can be observed in all age groups.^1^ It is characterized by tense clear or hemorrhagic blisters arising on normal skin or on an erythematous base, with a predilection for flexor surfaces. However, rare variants of BP that do not present with blistering also exist.^2^ BP is often accompanied by intense pruritus and undesirable changes to the physical appearance of the skin.^3^

BP has an autoimmune etiology, centred around the presence of autoantibodies against bullous pemphigoid antigen 1 (BP230), a cytoskeletal linker protein, and bullous pemphigoid antigen 2 (BP180), a component of collagen type XVII. While both BP180 and BP230 are strong predictors of BP onset and severity, BP180 is believed to play the primary role in pathogenesis.^4^ The pathogenesis of BP has not been fully elucidated, but is believed to involve a loss of self-tolerance in B- and T-cells and activation of toll-like receptors (TLRs), leading to production of autoantibodies against BP180 and/or BP230. Subsequent activation of complement, recruitment of granulocytes, release of pro-inflammatory cytokines, and matrix metalloproteases (MMPs) results in the breakdown of extracellular matrix and inflammatory infiltrate, forming the blisters observed in BP.^5^

Biomarkers are indicators of normal and pathogenic biological processes and can be used to measure response to external intervention.^6^ Applications of biomarkers include disease diagnosis, predicting prognosis, treatment selection, and use as an objective measure of treatment efficacy. Perhaps one of the most well-known examples of a diagnostic biomarker is hemoglobin A1c in type II diabetes mellitus (T2DM), which has revolutionized the diagnosis and prognosis of T2DM.^7^

The current gold standard for BP diagnosis is direct immunofluorescence visualization of linear immunoglobulin (Ig)G or C3 deposits at the dermoepidermal junction. However, sensitivity varies depending on biopsy site and there is a higher risk of false negatives in early-stage disease and in variants without blistering.^8^ While immunoserology for BP180 and BP230 autoantibodies can be used to support a diagnosis of BP, these autoantibodies can also be present in individuals without BP, raising the risk of false positives.^9^

Current standard management of BP involves the use of topical superpotent corticosteroids in mild/localized cases and oral corticosteroids in more severe/generalized cases. However, many patients relapse after stopping treatment, prompting prolonged treatment with corticosteroids which carries severe adverse effects, particularly in the elderly.^10^ Thus, effective steroid-sparing therapies are of need, particularly in more severe recalcitrant cases. With the potential for biomarkers to improve clinical management of BP and fill in gaps in our understanding of BP pathogenesis, the aim of this systematic review was to identify and categorize potentially relevant BP biomarkers into immune pathways.

## Materials and Methods

The literature search for this review (PROSPERO CRD42023466757) was conducted according to the Preferred Reporting Items for Systematic Reviews and Meta-analyses (PRISMA) guidelines.^11^ Medline and Embase were searched from inception to September 2023, with no exclusions on language or patient demographics. The removal of 281 duplicates yielded a total of 877 articles for screening. Full search strategy is provided in the Supplemental Material. A PRISMA flow diagram is provided (Supplemental Figure S1).

### Inclusion and Exclusion Criteria

All published articles reporting on serum biomarker levels of BP patients in comparison to healthy controls were included. Animal studies and articles that do not include healthy controls were excluded.

### Data Extraction

Articles were screened based on titles and abstract, followed by full-text screening to determine final eligibility. Article references were also evaluated for inclusion. EW and BW individually performed data extraction. Disagreements were mediated by meeting with a third author (RSQG) to reach a consensus. Data were analyzed with descriptive statistics. The National Institute of Health quality assessment tool was used to assess methodological quality, which provides quality ratings of “good,” “fair,” or “poor,” Only reports with a “good” rating were included.

### Data Analysis

Data were analyzed with descriptive statistics. Weighted associations were calculated by assigning studies that reported an increase of a biomarker in BP patients versus healthy controls a value of +1, no change in biomarkers a value of 0, and a decrease in biomarker levels a value of −1. Weighted associations for each biomarker were calculated based on patient sample size. Weighted association values >0.5 indicate most BP patients exhibit elevated levels of the biomarker compared to healthy controls, values between −0.5 and 0.5 indicate most BP patients do not exhibit differences in biomarker levels, and values <−0.5 indicate most BP patients exhibit lower levels of the biomarker. The specific inflammatory mediators involved in BP pathogenesis are currently unknown and there may be potential differences between individuals (especially for mediators that are a consequence of the pathology, rather than a contributor). This method of analyzing biomarker data allows for determining the overall trend in biomarker levels among different BP patients to identify which biomarkers are consistently elevated or reduced, as these may be the most informative and attractive as therapeutic targets.

## Results

A total of 877 unique articles were identified with the outlined search strategy. Of the 62 articles included, all were case-control studies, reporting on a total of 1837 patients with BP. One hundred and forty unique biomarkers were identified, categorized into T-cell mediated, B-cell mediated, innate immune system, and coagulation cascade pathways. A table summarizing all biomarkers identified in this study is provided (Supplemental Table S1).

### T_h_1 Biomarkers

Tumor necrosis factor (TNF)-α is a characteristic T_h_1 cytokine that was found to be elevated in BP patients, along with TNF-α dependent chemokines including c-x-c motif chemokine ligand 10.^12^ Other T_h_1 cytokines including interferon-γ, interleukin (IL)-2, among others, were largely unchanged in BP patients compared to healthy controls (Supplemental Table S1).

### T_h_2 Biomarkers

IL-4 is a characteristic T_h_2 cytokine that was found to be elevated in BP patients. In conjunction with TNF-a, IL-4 regulates the secretion of several other T_h_2 cytokines that were also found to be elevated, including c-c motif chemokine ligand (CCL) 17 and CCL22.^12^ Other T_h_2 cytokines including IL-10, IL-13, among others remained largely unchanged in BP patients compared to healthy controls (Supplemental Table S3).

### T_h_17 Biomarkers

Several T_h_17 cytokines including IL-16, IL-21, IL-26, and transforming growth factor (TGF)-β were found to be elevated in BP patients. However, the characteristic T_h_17 cytokine, IL-17, was most unchanged in BP patients compared to healthy controls (Supplemental Table S4).

### T_reg_ Biomarkers

With the exception of TGF-β, levels of all other T_reg_ cell biomarkers were largely unchanged in BP patients compared to healthy controls. However, one study did report an increase in T_reg_ cell count (Supplemental Table S5).^13^

### B-Cell Biomarkers and Antibodies

The pro-survival factor, B-cell activating factor (BAFF), and B_reg_ cell counts were noted to be elevated in BP patients (Supplemental Table S6). B_reg_ cells aid in suppressing immunopathology through the secretion of IL-10 and TGF-β.^14^

In terms of antibodies, elevations in anti-BP180/230 IgE and IgG autoantibodies, and total IgE levels were the most notable. There were no changes observed in total levels of IgG, IgD, or IgM (Supplemental Table S6).

### Innate Immune System Biomarkers

Within the innate immune system, elevations in eosinophils and eosinophil cationic protein (ECP), a marker of eosinophil activation, were the most notable (Supplemental Table S7). Elevations in MMP-9 and neutrophil enzymes, myeloperoxidase, were also noted.^15^

### Coagulation Cascade Biomarkers

There was consensus on the involvement of the coagulation cascade, with all data regarding coagulation cascade biomarkers were noted to be increased in BP patients (Supplemental Table S8).

## Discussion

To our knowledge, this is this first systematic review of reported serum biomarkers in BP. This review included 62 articles with a total of 1837 patients with BP, identifying 140 unique biomarkers. The biomarker trends identified in this study can potentially be used to develop an improved understanding of BP pathophysiology, identify drug targets for development, and inform on future studies to identify prognostic factors for diagnosis and guide treatment.

The pathogenesis of BP is multifactorial, with an underlying autoimmune etiology. Current theories revolve around three cross-communicating factors: an imbalance between T_reg_ and autoreactive T_h_ cells, TLR activation and T_h_17 cell activation, that leads to the production of autoantibodies against BP180 and/or BP230, and increased, neutrophil, eosinophil, T_h_2 cell, complement and coagulation cascade activity. The resulting release of pro-inflammatory cytokines and degradative enzymes ultimately results in the inflammation, pruritus, and blister formation that is characteristic of BP.^16,17^ A diagram illustrating the pathogenesis pathway is provided (Supplemental Figure S1).

While several key components of the pathogenesis pathways were supported by the biomarker trends identified in our study, it is important to recognize that the present study only includes serum biomarkers, which may not fully represent the pathogenic processes occurring locally in the skin. There was consensus among the studies reporting on anti-BP180/230 IgG/E and total IgE levels in serum, with increases noted (Supplemental Table S6). In terms of T_h_17 cell activity, no change in IL-17 was observed in serum (Supplemental Table S3). However, studies reporting on IL-17 in blister fluid have noted increases.^18,19^ While a mixed T_h_1/T_h_2 response was originally believed to be involved in BP pathogenesis, a greater focus has been placed on T_h_2 cells due to their role in promoting B-cell proliferation and antibody production via IL-4, and promoting eosinophil activity via IL-4/5.^20,21^ In our study, most reported BP patients had elevations in IL-4/5 (Supplemental Table S3). Further support for T_h_2 involvement is provided by an increase in IL-31 in serum and skin, which was notable as IL-31 is a potent driver of pruritic symptoms, which is usually present in BP (Supplemental Table S3).^22^ In terms of T_reg_ cell activity, an increased level of T_reg_ cell count was observed in BP patients (Supplemental Table S5). This suggests that even an increase number of T_reg_ cells is insufficient to inhibit the inflammation, indicating an imbalance between pro-inflammatory and regulatory T-cells. In terms of the innate immune system, the results support the role of eosinophils in BP pathogenesis. With the exception of one, all studies reported increased eosinophil cell count and ECP. Neutrophil myeloperoxidase and MMP-9 levels were also elevated, potentially contributing to inflammatory tissue damage. (Supplemental Tables S7 and S8).

With the current diagnostic framework of performing direct immunofluorescence and indirect immunofluorescence ± a BP180 enzyme-linked immunosorbent assay, a >98% detection of BP can be achieved.^8^ Given the high accuracy rate, it may not be necessary to include a biomarker assay in the initial workup. Furthermore, the biomarkers included in this study (with the exception of BP180/230 autoantibodies) have not been verified to be correlated with BP onset, severity, remission, or treatment outcomes. Thus, as of now, the use of biomarker panels for diagnosis may not provide additional diagnostic value. However, the biomarker trends identified may be used to inform future studies in identifying prognostic factors for diagnosis and guiding treatment. This could potentially allow for patients with confirmed BP recalcitrant to standard therapy to be assessed with a biomarker panel to identify specific inflammatory mediators for targeted inhibition. This would permit tailoring therapeutic regimens to the individual patient and potentially avoiding adverse reactions associated with prolonged use of nonspecific immunosuppressants such as corticosteroids.

Current management of BP revolves around the use of corticosteroids, often in combination with other immunosuppressants including azathioprine and mycophenolate mofetil.^23^ However, many patients relapse after tapering corticosteroids, prompting unfavorable prolonged corticosteroid therapy.^10^ Due to the chronic and often recalcitrant nature of BP, expanded treatment options, including targeted treatment, are needed for this challenging to treat population. Given the autoimmune etiology centred around production of anti-BP180/230 autoantibodies, B-cell inhibitors should provide efficacy in treating BP. In a retrospective cohort study, 75% of BP patients treated with rituximab achieved remission and demonstrated steroid-sparing activity.^24^ However, patients treated with rituximab may experience disease flares after treatment cessation, as observed in systemic lupus erythematous patients. This may be due to increased levels of the B-cell pro-survival factor, BAFF, promoting B-cell proliferation after cessation of treatment. As such, treatment with rituximab followed by a BAFF inhibitor (eg, belimumab) may provide more robust remission in BP patients. The efficacy of such a treatment regimen has been demonstrated in a case report of a patient with BP that was refractory to conventional therapy successfully treated with sequential rituximab and belimumab.^25^ However, larger studies are needed to further investigate efficacy and safety.

Due to the role of T_h_2 activity in promoting B-cell proliferation, inhibition of T_h_2 pathways may also be efficacious in treating BP. In a retrospective cohort study, 87% of BP patients treated with dupilumab off-label (IL-4/13 inhibitor) achieved disease control within 4 weeks, with improvement in anti-BP180/230 autoantibody levels, total IgE, and eosinophil levels.^26^ TNF-α works synergistically with IL-4 to increase secretion of T_h_2 cytokines, suggesting potential efficacy in BP by targeting TNF-α.^12^ Indeed, cases of BP successfully treated with etanercept have been reported.^27^ However, cases of BP induced by use of TNF-α inhibitors (eg, etanercept, infliximab) have also been reported.^25^ The underlying mechanism is unclear, but may involve increased cell apoptosis post etanercept treatment resulting in increased autoantigen exposure and autoantibody production.^28^ Thus, additional studies are required to assess the efficacy of TNF-α inhibitors in treating BP, particularly in BP patients that exhibit elevated levels of TNF-α and IL-4.

Limitations of our study include small sample sizes in individual studies, heterogenous patient population, and the use of different laboratory assays to assess various biomarkers. The present study only included articles that studied levels of serum biomarkers in BP patients in comparison to healthy controls. This may have resulted in the exclusion of some relevant biomarkers, including complement pathway components known to be involved in BP pathogenesis. An additional limitation is the assessment of only serum biomarkers, which may not accurately reflect the pathogenic processes occurring locally in the skin, especially in less generalized cases of BP. Future studies are required to compare the biomarker levels in serum with blister fluid and skin biopsies to differentiate between local and systemic pathogenic processes.

## Conclusion

BP is the most common type of subepidermal blistering disease, with significant psychosocial impacts.^1,3^ Serum biomarkers of BP identified in this study, including anti-BP180/230 IgG/E, total IgE, IL-31, BAFF, TNF-α, ECP, MMP-9, and various coagulation cascade biomarkers support the current theories of BP pathogenesis summarized in Supplemental Figure S1. The biomarker trends identified in this systematic review can potentially be used to develop an improved understanding of BP pathophysiology, identify drug targets for development, and inform on future studies to identify prognostic factors for diagnosis and guide treatment.

## Supplemental Material

sj-docx-1-cms-10.1177_12034754241266171 – Supplemental material for Serum Biomarkers in Bullous Pemphigoid: A Systematic Review Supplemental material, sj-docx-1-cms-10.1177_12034754241266171 for Serum Biomarkers in Bullous Pemphigoid: A Systematic Review by Ryan S. Q. Geng, Elizabeth Wei, Bethany Wilken, Ronald G. Sibbald and Cathryn Sibbald in Journal of Cutaneous Medicine and Surgery

sj-docx-2-cms-10.1177_12034754241266171 – Supplemental material for Serum Biomarkers in Bullous Pemphigoid: A Systematic Review Supplemental material, sj-docx-2-cms-10.1177_12034754241266171 for Serum Biomarkers in Bullous Pemphigoid: A Systematic Review by Ryan S. Q. Geng, Elizabeth Wei, Bethany Wilken, Ronald G. Sibbald and Cathryn Sibbald in Journal of Cutaneous Medicine and Surgery
