# Supplementary material for: Serum Biomarkers in Bullous Pemphigoid: A Systematic Review
Source: J Cutan Med Surg. 2024 Jul 29;28(5):463–7. doi: 10.1177/12034754241266171 (PMC11514321; doi:10.1177/12034754241266171)
Supplement: sj-docx-1-cms-10.1177_12034754241266171 – Supplemental material for Serum Biomarkers in Bullous Pemphigoid: A Systematic Review [file sj-docx-1-cms-10.1177_12034754241266171.docx]

| **Article**  **Table S1.** BP biomarkers reported in the studies included in this review. | **N patients (mean age)** | **N controls (mean age)** | **Biomarker** | **Biomarker type, medium** | **Value in BP patients**  **(Mean ± SD)** | **Value in controls**  **(Mean ± SD)** | **Overall association** |
| --- | --- | --- | --- | --- | --- | --- | --- |
| Asano 2008^1^ | 21 (72) | 45 (45) | MIF | Protein, Serum | 24.0 **±** 1.94 | 4.1 **±** 0.26 | Increase |
| Asashima 2006^2^ | 39 (74) | 22 (70) | BAFF | Protein, Serum | 6.62 ± 2.82 | 4.44 ± 0.77 | Increase |
| Bieber 2017^3^ | 25 (73) | 56 (73) | ECP | Protein, Serum | NR | NR | Increase |
|  | 25 (73) | 56 (73) | CD4 | Protein, Serum | NR | NR | Increase |
|  | 25 (73) | 56 (73) | IgD | Protein, Serum | NR | NR | No change |
|  | 25 (73) | 56 (73) | IL-2R | Protein, Serum | NR | NR | No change |
|  | 25 (73) | 56 (73) | L-selectin | Protein, Serum | NR | NR | No change |
|  | 25 (73) | 56 (73) | MCT | Protein, Serum | NR | NR | No change |
|  | 25 (73) | 56 (73) | MPO | Protein, Serum | NR | NR | Increase |
|  | 25 (73) | 56 (73) | Neopterin | Protein, Serum | NR | NR | No change |
|  | 25 (73) | 56 (73) | P-selectin | Protein, Serum | NR | NR | Increase |
|  | 25 (73) | 56 (73) | S100A12 | Protein, Serum | NR | NR | Increase |
| Bornscheuer 1999^4^ | 19 (NR) | 5 (NR) | CCL-5 | Protein, Serum | NR | NR | No change |
| Czech 1993^5^ | 12 (68) | 12 (35) | ECP | Protein, Serum | 60.5 | 8.8 | Increase |
|  | 12 (68) | 12 (35) | IgE | Protein, Serum | 420 | 45.1 | Increase |
|  | 12 (68) | 12 (35) | MPO | Protein, Serum | 748 | 368 | Increase |
|  | 12 (68) | 12 (35) | MPO/ECP ratio | Protein, Serum | 26.9 | 54.1 | Decrease |
| D'Auria 1998^6^ | 15 (82) | 15 (76) | CCL-5 | Protein, Serum | 35,822 | 38,697 | No change |
|  | 15 (82) | 15 (76) | ECP | Protein, Serum | 12.6 | 5.6 | No change |
|  | 15 (82) | 15 (76) | IgE | Protein, Serum | 344 | 23 | Increase |
|  | 15 (82) | 15 (76) | IL-5 | Protein, Serum | 0.1 | 0.1 | No change |
| D'Auria 1999^7^ | 15 (82) | 20 (73)) | IL-10 | Protein, Serum | 2.7 | 2 | Increase |
|  | 15 (82) | 20 (73)) | IL-6 | Protein, Serum | 17.8 | 1.6 | Increase |
|  | 15 (82) | 20 (73)) | TNF-α | Protein, Serum | 5.3 | 4.4 | Increase |
| D'Auria 2000^8^ | 15 (84) | 15 (81) | MPO | Protein, Serum | 509 | 324 | No change |
|  | 15 (84) | 15 (81) | Tryptase | Protein, Serum | 0.5 | 0.5 | No change |
| De Pita 1997^9^ | 25 (NR) | 10 (NR) | CD30 | Protein, Serum | 30.3 | 10.5 | Increase |
|  | 25 (NR) | 10 (NR) | IL-4 | Protein, Serum | 16.6 ± 7.9 | 4.5 ± 2.2 | Increase |
| Dimson 2003^10^ | 30 (NR) | 17 (NR) | IgE | Protein, Serum | 399 | 31 | Increase |
| Echigo 2006^11^ | 27 (67) | 20 (NR) | CCL-17 | Protein, Serum | 1,026 ± 1,798.5 | 93.3 ± 25.3 | Increase |
|  | 27 (67) | 20 (NR) | CCL-22 | Protein, Serum | 2,003.4 ± 2,892.8 | 155.8 ± 61 | Increase |
|  | 27 (67) | 20 (NR) | CXCL-9 | Protein, Serum | 17.1 ± 15.1 | 3.7 ± 2.6 | Increase |
| Echigo 2007^12^ | 28 (69) | 32 (60) | Anti-cardiolipin IgG | Protein, Serum | NR | NR | Increase |
| Fang 2023^13^ | 15 (NR) | 13 (NR) | CXCR4 | mRNA, serum | NR | NR | Increase |
| Freire 2017^14^ | 19 (NR) | 18 (NR) | Anti-BP180 IgE | Protein, Serum | NR | NR | Increase |
|  | 19 (NR) | 18 (NR) | Anti-BP230 IgE | Protein, Serum | NR | NR | Increase |
| Frezzolini 2004^15^ | 39 (NR) | 10 (NR) | CD30 | Protein, Serum | 31 ± 18 | 10 ± 3 | Increase |
|  | 39 (NR) | 10 (NR) | ECP | Protein, Serum | 17 ± 21 | 5 ± 7 | Increase |
|  | 39 (NR) | 10 (NR) | IL-16 | Protein, Serum | 680 ± 433 | 287 ± 72 | Increase |
| Fujimura 2017^16^ | 10 (NR) | NR | CD163 | Protein, Serum | NR | NR | Increase |
|  | 10 (NR) | NR | CXCL-5 | Protein, Serum | NR | NR | No change |
| Furukawa 1994^17^ | 13 (NR) | 20 (NR) | CD23 | Protein, Serum | 17.8 ± 6.5 | 8.1 ± 1.5 | No change |
| Giacalone 1998^18^ | 18 (82) | 18 (73) | IL-7 | Protein, Serum | 5.98 | 2.74 | Increase |
|  | 18 (82) | 18 (73) | TGF-β | Protein, Serum | 37.0 | 36.6 | No change |
| Gunther 2009^19^ | 14 (NR) | 12 (NR) | CCL-18 | Protein, Serum | NR | NR | Increase |
| Gunther 2011^20^ | 25 (77) | 43 (38) | CCL-11 | Protein, Serum | 270 ± 103 | 48 ± 79 | Increase |
|  | 25 (77) | 43 (38) | CCL-24 | Protein, Serum | 310 ± 204 | 274 ± 293 | No change |
|  | 25 (77) | 43 (38) | CCL-26 | Protein, Serum | NR | NR | Increase |
| Hatano 2003^21^ | 1 (58) | 2 (NR) | CCL-3 | mRNA, Serum | NR | NR | Increase |
|  | 1 (58) | 2 (NR) | IFN-γ | mRNA, Serum | NR | NR | Increase |
|  | 1 (58) | 2 (NR) | IL-4 | mRNA, Serum | NR | NR | No change |
|  | 1 (58) | 2 (NR) | IL-5 | mRNA, Serum | NR | NR | Increase |
|  | 1 (58) | 2 (NR) | IL-8 | mRNA, Serum | NR | NR | Increase |
| Hu 2022^22^ | 10 (NR) | 15 (NR) | CCL-17 | Protein, Serum | NR | NR | Increase |
|  | 10 (NR) | 15 (NR) | IFN-γ | Protein, Serum | NR | NR | No change |
|  | 10 (NR) | 15 (NR) | IL-22 | Protein, Serum | NR | NR | Increase |
|  | 10 (NR) | 15 (NR) | IL-6 | Protein, Serum | NR | NR | Increase |
|  | 10 (NR) | 15 (NR) | TSLP | Protein, Serum | 43.22 | 37.67 | No change |
| Inaoki 1998^23^ | 19 (75) | 16 (66) | CD23 | Protein, Serum | 119 ± 152 | 99 ± 70 | No change |
|  | 19 (75) | 16 (66) | IL-13 | Protein, Serum | 19 ± 31 | 17 ± 36 | No change |
|  | 19 (75) | 16 (66) | IL-2 | Protein, Serum | 69 ± 105 | 50 ± 73 | No change |
|  | 19 (75) | 16 (66) | IL-2R | Protein, Serum | 61 ± 36 | 53 ± 24 | No change |
|  | 19 (75) | 16 (66) | IL-4 | Protein, Serum | 53 ± 91 | 108 ± 191 | No change |
|  | 19 (75) | 16 (66) | IL-5 | Protein, Serum | 41 ± 21 | 7 ± 9 | Increase |
|  | 19 (75) | 16 (66) | IL-6 | Protein, Serum | 193 ± 334 | 25 ± 52 | Increase |
|  | 19 (75) | 16 (66) | IL-8 | Protein, Serum | 110 ± 187 | 6 ± 13 | Increase |
| Inaoki 2006^24^ | 28 (76) | 22 (71) | CD20 | Protein, Serum | NR | NR | No change |
|  | 28 (76) | 22 (71) | CD22 | Protein, Serum | NR | NR | Decrease |
|  | 28 (76) | 22 (71) | CD40 | Protein, Serum | NR | NR | No change |
|  | 28 (76) | 22 (71) | IgE | Protein, Serum | 894 ± 1338 | 212 ± 313 | Increase |
|  | 28 (76) | 22 (71) | IgG | Protein, Serum | 1,329 ± 482 | 1,413 ± 279 | No change |
|  | 28 (76) | 22 (71) | IgM | Protein, Serum | 85.2 ± 45.0 | 91.0 ± 39.8 | No change |
| Julio 2018^25^ | 50 (NR) | 50 (NR) | BDNF | Protein, Serum | 16.88 | 38.21 | Decrease |
| Kakinuma 2003^26^ | 13 (NR) | 8 (NR) | CCL-17 | Protein, Serum | 1,151.5 ± 885.6 | 196.6 ± 129.7 | Increase |
| Khil'chenko 2020^27^ | 99 (NR) | 105 (NR) | IgA | Protein, Serum | 0.34 | NR | Increase |
|  | 99 (NR) | 105 (NR) | IgG | Protein, Serum | NR | NR | No change |
|  | 99 (NR) | 105 (NR) | IgG1 | Protein, Serum | NR | NR | Increase |
|  | 99 (NR) | 105 (NR) | IgG2 | Protein, Serum | 0.64 | 1.05 | Decrease |
|  | 99 (NR) | 105 (NR) | IgG4 | Protein, Serum | NR | NR | Increase |
|  | 99 (NR) | 105 (NR) | IgM | Protein, Serum | NR | 0.56 | No change |
| Kieffer 1983^28^ | 34 (NR) | 23 (NR) | IgA | Protein, Serum | NR | NR | Increase |
|  | 34 (NR) | 23 (NR) | IgD | Protein, Serum | NR | NR | No change |
|  | 34 (NR) | 23 (NR) | IgG | Protein, Serum | NR | NR | Increase |
|  | 34 (NR) | 23 (NR) | IgM | Protein, Serum | NR | NR | No change |
| Kokubu 2023^29^ | 39 (77) | 10 (42) | IL-10 | Protein, Serum | 7.63 ± 5.03 | 6.88 ± 0.52 | No change |
|  | 39 (77) | 10 (42) | IL-35 | Protein, Serum | 2.62 ± 0.20 | 2.60 ± 0.17 | No change |
| Le Jan 2014^30^ | 31 (NR) | NR | IL-1β | Protein, Serum | 0.046 ± 0.024 | 0.178 ± 0.107 | No change |
|  | 31 (NR) | NR | IL-17 | Protein, Serum | NR | NR | No change |
|  | 31 (NR) | NR | IL-22 | Protein, Serum | NR | NR | No change |
|  | 31 (NR) | NR | IL-23 | Protein, Serum | NR | NR | No change |
|  | 31 (NR) | NR | IL-6 | Protein, Serum | NR | NR | No change |
|  | 31 (NR) | NR | MMP-9 | Protein, Serum | NR | NR | Increase |
|  | 31 (NR) | NR | TGF-β | Protein, Serum | NR | NR | Increase |
| Le Jan 2019^31^ | 71 (82) | NR | IL-1β | Protein, Serum | 0.046 ± 0.024 | 0.178 ± 0.107 | No change |
| Li 2013^32^ | 32 (NR) | 14 (NR) | IL-21 | Protein, Serum | 103.98 | 46.77 | Increase |
| Liu 2017^33^ | 16 (NR) | 20 (NR) | TWEAK | Protein, Serum | NR | NR | Increase |
| Liu 2018^34^ | 25 (NR) | 25 (NR) | B-reg cells | Cell, Serum | NR | NR | Increase |
|  | 25 (NR) | 25 (NR) | IFN-γ | mRNA, Serum | NR | NR | Increase |
|  | 25 (NR) | 25 (NR) | IL-10 | mRNA, Serum | NR | NR | No change |
|  | 25 (NR) | 25 (NR) | IL-22 | mRNA, Serum | NR | NR | No change |
|  | 25 (NR) | 25 (NR) | IL-23 | mRNA, Serum | NR | NR | No change |
|  | 25 (NR) | 25 (NR) | IL-6 | mRNA, Serum | NR | NR | No change |
|  | 25 (NR) | 25 (NR) | TNF-α | mRNA, Serum | NR | NR | Increase |
| Maglie 2023^35^ | 34 (75) | 25 (68) | IL-26α | Protein, Serum | NR | NR | Increase |
|  | 34 (75) | 25 (68) | IL-26β | Protein, Serum | NR | NR | Increase |
|  | 34 (75) | 25 (68) | IL-26γ | Protein, Serum | NR | NR | Increase |
|  | 34 (75) | 25 (68) | IL-36Ra | Protein, Serum | NR | NR | No change |
|  | 34 (75) | 25 (68) | IL-38 | Protein, Serum | NR | NR | No change |
| Marzano 2011^36^ | 63 (75) | 40 (50) | D-dimer | Protein, Serum | 2,285 ± 3,010 | 190 ± 99 | Increase |
|  | 63 (75) | 40 (50) | Prothrombin fragment F1+2 | Protein, Serum | 500 ± 377 | 109 ± 38 | Increase |
| Messingham 2014^37^ | 48 (78) | 58 (80) | Anti-BP180 IgG | Protein, Serum | NR | NR | Increase |
|  | 48 (78) | 58 (80) | Anti-BP230 IgG | Protein, Serum | NR | NR | Increase |
|  | 48 (78) | 58 (80) | IgE | Protein, Serum | NR | NR | Increase |
| Muramatsu 2020^38^ | 18 (71) | 31 (NR) | Treg cells | Cell, Serum | NR | NR | Increase |
| Nakashima 2007^39^ | 38 (71) | 17 (62) | CCL-11 | Protein, Serum | NR | NR | No change |
|  | 38 (71) | 17 (62) | CCL-2 | Protein, Serum | NR | NR | Increase |
|  | 38 (71) | 17 (62) | CCL-3 | Protein, Serum | NR | NR | No change |
|  | 38 (71) | 17 (62) | CCL-4 | Protein, Serum | NR | NR | No change |
|  | 38 (71) | 17 (62) | CCL-5 | Protein, Serum | NR | NR | No change |
|  | 38 (71) | 17 (62) | CCL-7 | Protein, Serum | NR | NR | No change |
|  | 38 (71) | 17 (62) | CCL-8 | Protein, Serum | NR | NR | No change |
|  | 38 (71) | 17 (62) | CXCL-1 | Protein, Serum | NR | NR | No change |
|  | 38 (71) | 17 (62) | CXCL-10 | Protein, Serum | NR | NR | Increase |
|  | 38 (71) | 17 (62) | CXCL-9 | Protein, Serum | NR | NR | No change |
| Nesmond 2019^40^ | 83 (81) | 46 (NR) | IL-17A | Protein, Serum | NR | NR | No change |
|  | 83 (81) | 46 (NR) | IL-17A/F | Proteinm, Serum | NR | NR | No change |
|  | 83 (81) | 46 (NR) | IL-17B | Protein, Serum | NR | NR | No change |
|  | 83 (81) | 46 (NR) | IL-17E | Protein, Serum | NR | NR | No change |
| Pruessmann 2021^41^ | 40 (80) | 40 (79) | Galactin-9 | Protein, Serum | 2.65 ± 1.02 | 1.95 ± 1.18 | Increase |
| Qian 2014^42^ | 11 (NR) | 7 (NR) | BAFF | Protein, Serum | NR | NR | Increase |
| Qiao 2017^43^ | 36 (NR) | 16 (NR) | CD46 | Protein, Serum | 139.50 ± 28.21 | 36.26 ± 15.68 | Increase |
| Qiu 2020^44^ | 45 (73) | 60 (49) | CCL-17 | Protein, Serum | 1,368 | 248.5 | Increase |
|  | 45 (73) | 60 (49) | miR-1291 | miRNA, Serum | 0.31 | 0.06 | Increase |
|  | 45 (73) | 60 (49) | miR-27a-5p | miRNA, Serum | 0.19 | 0.08 | Increase |
|  | 45 (73) | 60 (49) | miR-423-5p | miRNA, Serum | 3.09 | 1.73 | Increase |
| Riani 2017^45^ | 114 (81) | 34 (NR) | CXCL-10 | Protein, Serum | 162 ± 21 | 67 ± 10 | Increase |
| Riani 2019^46^ | 12 (NR) | 8 (NR) | Arginase | Protein, Serum | NR | NR | No change |
|  | 12 (NR) | 8 (NR) | CCL-17 | Protein, Serum | NR | NR | No change |
|  | 12 (NR) | 8 (NR) | IFN-γ | Protein, Serum | NR | NR | No change |
|  | 12 (NR) | 8 (NR) | IL-10 | Protein, Serum | NR | NR | No change |
|  | 12 (NR) | 8 (NR) | IL-1RA | Protein, Serum | NR | NR | No change |
|  | 12 (NR) | 8 (NR) | TNF-α | Protein, Serum | NR | NR | No change |
| Rifaioglu 2014^47^ | 19 (68) | 22 (62) | C reactive protein | Protein, Serum | 1.201 +/- 1.113 | 1.342 +/- 1.430 | No change |
|  | 19 (68) | 22 (62) | Eosinophils | Cell, Serum | 1.03 +/- 0.36 x 10^3 | 0.22 +/- 0.29 x 10^3 | Increase |
|  | 19 (68) | 22 (62) | ESR | N/A | 19.58 +/- 12.98 | 14.32 +/- 8.84 | No change |
|  | 19 (68) | 22 (62) | MPV | N/A | 8.32 +/- 1.8 | 7.24 +/- 0.7 | Increase |
|  | 19 (68) | 22 (62) | Platelets | Cell, Serum | 241.38 +/- 9.95 x 10^3 | 267.82 +/- 6.53 x 10^3 | No change |
|  | 19 (68) | 22 (62) | WBC | Cell, Serum | 8.86 +/- 3.71 x 10^3 | 7.83 +/- 1.33 x 10^3 | No change |
| Rudrich 2018^48^ | 22 (82) | 11 (NR) | IL-31 | Protein, Serum | NR | NR | Increase |
| Salz 2017^49^ | 64 (NR) | 19 (NR) | IL-31 | Protein, Serum | 6.9 | NR | Increase |
| Schaller 1995^50^ | 15 (72) | 26 (67) | CD23R | Protein, Serum | 5.5 +/- 1.8 | 0.5 +/- 0.02 | No change |
|  | 15 (72) | 26 (67) | IFN-γ | Protein, Serum | 0.8 +/- 0.4 | 3.6 +/- 1.3 | No change |
|  | 15 (72) | 26 (67) | IgE | Protein, Serum | 524 +/- 110.1 | 11.8 +/- 3.0 | Increase |
|  | 15 (72) | 26 (67) | IL-2R | Protein, Serum | 177.3 +/- 29.0 | 85.9 +/- 7.1 | Increase |
|  | 15 (72) | 26 (67) | IL-4 | Protein, Serum | 5.9 +/- 3.5 | 0.9 +/- 0.2 | No change |
| Shen 2018^51^ | 32 (NR) | 11 (NR) | CD100 | Protein, Serum | NR | NR | Increase |
| Shirakata 1990^52^ | 15 (NR) | 10 (NR) | IgG | Protein, Serum | 12.9 +/- 4.8 | 12.7 +/- 2.7 | No change |
|  | 15 (NR) | 10 (NR) | IgG1 | Protein, Serum | 6.61 +/- 3.96 | 5.88 +/- 1.78 | No change |
|  | 15 (NR) | 10 (NR) | IgG2 | Protein, Serum | 3.08 +/- 2.48 | 3.42 +/- 1.41 | No change |
|  | 15 (NR) | 10 (NR) | IgG3 | Protein, Serum | 0.40 +/- 0.16 | 0.93 +/- 0.30 | Decrease |
|  | 15 (NR) | 10 (NR) | IgG4 | Protein, Serum | 3.81 +/- 1.81 | 1.14 +/- 0.39 | Increase |
| Shrikhande 2000^53^ | 6 (74) | 6 (74) | ECP | Protein, Serum | 29 +/- 7.9 | 5.6 +/- 1.2 | Increase |
|  | 6 (74) | 6 (74) | Eosinophils | Cell, Serum | 1.1 +/- 0.38 x 10^9 | 0.1 +/- 0.04 x 10^9 | Increase |
| Suzuki 2021^54^ | 20 (NR) | 24 (NR) | CCL-3 | Protein, Serum | NR | NR | Decrease |
|  | 20 (NR) | 24 (NR) | CCL-4 | Protein, Serum | NR | NR | Decrease |
|  | 20 (NR) | 24 (NR) | CXCL-10 | Protein, Serum | NR | NR | Decrease |
|  | 20 (NR) | 24 (NR) | FGF-basic | Protein, Serum | NR | NR | Decrease |
|  | 20 (NR) | 24 (NR) | G-CSF | Protein, Serum | NR | NR | Decrease |
|  | 20 (NR) | 24 (NR) | GM-CSF | Protein, Serum | NR | NR | Decrease |
|  | 20 (NR) | 24 (NR) | IFN-γ | Protein, Serum | NR | NR | Decrease |
|  | 20 (NR) | 24 (NR) | IL-13 | Protein, Serum | NR | NR | Decrease |
|  | 20 (NR) | 24 (NR) | IL-17 | Protein, Serum | NR | NR | Increase |
|  | 20 (NR) | 24 (NR) | IL-7 | Protein, Serum | NR | NR | Decrease |
|  | 20 (NR) | 24 (NR) | IL-8 | Protein, Serum | NR | NR | Decrease |
|  | 20 (NR) | 24 (NR) | IL-9 | Protein, Serum | NR | NR | Increase |
|  | 20 (NR) | 24 (NR) | PDGF-BB | Protein, Serum | NR | NR | Decrease |
|  | 20 (NR) | 24 (NR) | TNF-α | Protein, Serum | NR | NR | Increase |
|  | 20 (NR) | 24 (NR) | VEGF | Protein, Serum | NR | NR | Decrease |
| Tedeschi 2015^55^ | 30 (78) | 30 (NR) | D-dimer | Protein, Serum | 4,262 +/- 686 | 249.3 +/- 14.9 | Increase |
|  | 30 (78) | 30 (NR) | ECP | Protein, Serum | 20.0 +/- 7.8 | 2.3 +/- 0.1 | Increase |
|  | 30 (78) | 30 (NR) | Prothrombin fragment F1+2 | Protein, Serum | 780.0 +/- 76.8 | 151.8 +/- 9.1 | Increase |
| Teraki 2001^56^ | 8 (NR) | 11 (NR) | CLA | Protein, Serum | 13.8 +/- 1.1 | NR | No change |
|  | 8 (NR) | 11 (NR) | IFN-γ | Protein, Serum | 35.8 +/- 6.3 | 18.1 +/- 2.0 | Increase |
|  | 8 (NR) | 11 (NR) | IL-10 | Protein, Serum | NR | NR | No change |
|  | 8 (NR) | 11 (NR) | IL-13 | Protein, Serum | 3.9 +/- 0.7 | 3.9 +/- 0.7 | Increase |
|  | 8 (NR) | 11 (NR) | IL-2 | Protein, Serum | NR | NR | No change |
|  | 8 (NR) | 11 (NR) | IL-4 | Protein, Serum | 4.2 +/- 0.8 | 1.7 +/- 0.2 | Increase |
|  | 8 (NR) | 11 (NR) | TNF-α | Protein, Serum | 70.0 +/- 3.1 | 40.5 +/- 6.6 | Increase |
| Treco 2023^57^ | 26 (73) | 26 (75) | Anti-BP180 IgE | Protein, Serum | NR | NR | Increase |
|  | 26 (73) | 26 (75) | CD48 | Protein, Serum | NR | NR | Increase |
|  | 26 (73) | 26 (75) | IgE | Protein, Serum | NR | NR | Increase |
| Watanabe 2007^58^ | 35 (71) | 12 (54) | CD40L | Protein, Serum | 4.17 | 2.02 | Increase |
| Watanabe 2007^59^ | 43 (70) | 15 (53) | TNFSF13 | Protein, Serum | 20.31 | 7.84 | Increase |
| Zebrowska 2009^60^ | 14 (69) | 10 (42) | CCL-11 | Protein, Serum | 157.9 | 119.4 | No change |
|  | 14 (69) | 10 (42) | CCL-17 | Protein, Serum | 249.4 | 158.6 | No change |
|  | 14 (69) | 10 (42) | CCL-2 | Protein, Serum | 368.0 | NR | No change |
| Zebrowska 2014^61^ | 27 (69) | 20 (72) | Chymase | Protein, Serum | 34.05 +/- 0.21 | 32.10 +/- 0.82 | No change |
|  | 27 (69) | 20 (72) | IL-4 | Protein, Serum | 68.66 +/- 6.65 | 66.98 +/- 10.85 | No change |
|  | 27 (69) | 20 (72) | MMP-9 | Protein, Serum | 364.79 +/- 41.38 | 52.61 +/- 0.78 | Increase |
|  | 27 (69) | 20 (72) | PAF | Protein, Serum | 218.19 +/- 29.87 | 29.87 +/- 2.97 | Increase |
|  | 27 (69) | 20 (72) | TNF-α | Protein, Serum | 57.26 +/- 1.26 | 62.11 +/- 2.56 | No change |
|  | 27 (69) | 20 (72) | Tryptase | Protein, Serum | 19.79 +/- 1.98 | 19.33 +/- 3.23 | No change |
| Zebrowska 2015^62^ | 27 (69) | 20 (72) | D-dimer | Protein, Serum | 0.49 +/- 0.4 | 0.29 +/- 0.3 | Increase |
|  | 27 (69) | 20 (72) | Fibrinogen | Protein, Serum | 4.50 +/- 0.18 | 2.60 +/- 0.3 | Increase |
|  | 27 (69) | 20 (72) | Tissue factor | Protein, Serum | NR | NR | Increase |

Abbreviations: B-cell activating factor belonging to the tumor necrosis factor family (BAFF), Brain-derived neurotrophic factor (BDNF), c-c motif chemokine ligand (CCL), cluster of differentiation (CD), cutaneous lymphocyte-associated antigen (CLA), c-x-c motif chemokine ligand (CXCL), eosinophil cationic protein (ECP), erythrocyte sedimentation rate (ESR), fibroblast growth factor (FGF), granulocyte colony stimulating factor (G-CSF), granulocyte-macrophage colony stimulating factor (GM-CSF), intercellular adhesion molecule (ICAM), interferon (IFN), interleukin (IL), Janus kinase (JAK), mast cell tryptase (MCT), macrophage derived chemokine (MDC), macrophage migratory inhibitory factor (MIF), matrix metalloprotease (MMP), myeloperoxidase (MPO), mean platelet volume (MPV), platelet activating factor (PAF), platelet derived growth factor subunit B (PDGF-BB), Signal transducer and activator of transcription (STAT), transforming growth factor (TGF), tumor necrosis factor (TNF), tumor necrosis factor superfamily member 13 (TNFSF13), tissue plasminogen activator (tPA), thymic stromal lymphopoietin (TSLP), tumor necrosis factor-like weak inducer of apoptosis (TWEAK), urokinase (uPA), vascular cell adhesion molecule (VCAM), vascular endothelial growth factor (VEGF), white blood cells (WBC).

**References**

1. Asano Y, Makino T, Norisugi O, Watanabe H, Abe R, Shimizu H, et al. Macrophage migration inhibitory factor (MIF) in bullous pemphigoid. J Dermatol Sci. 2008;49(1):95-7.

2. Asashima N, Fujimoto M, Watanabe R, Nakashima H, Yazawa N, Okochi H, et al. Serum levels of BAFF are increased in bullous pemphigoid but not in pemphigus vulgaris. Br J Dermatol. 2006;155(2):330-6.

3. Bieber K, Ernst AL, Tukaj S, Holtsche MM, Schmidt E, Zillikens D, et al. Analysis of serum markers of cellular immune activation in patients with bullous pemphigoid. Exp Dermatol. 2017;26(12):1248-52.

4. Bornscheuer E, Zillikens D, Schroder JM, Sticherling M. Lack of expression of interleukin 8 and RANTES in autoimmune bullous skin diseases. Dermatology. 1999;198(2):118-21.

5. Czech W, Schaller J, Schopf E, Kapp A. Granulocyte activation in bullous diseases: Release of granular proteins in bullous pemphigoid and pemphigus vulgaris. Journal of the American Academy of Dermatology. 1993;29(2 I):210-5.

6. D'Auria L, Pietravalle M, Mastroianni A, Ferraro C, Mussi A, Bonifati C, et al. IL-5 levels in the serum and blister fluid of patients with bullous pemphigoid: Correlations with eosinophil cationic protein, RANTES, IgE and disease severity. Archives of Dermatological Research. 1998;290(1-2):25-7.

7. D'Auria L, Mussi A, Bonifati C, Mastroianni A, Giacalone B, Ameglio F. Increased serum IL-6, TNF-alpha and IL-10 levels in patients with bullous pemphigoid: Relationships with disease activity. Journal of the European Academy of Dermatology and Venereology. 1999;12(1):11-5.

8. D'Auria L, Pietravalle M, Cordiali-Fei P, Ameglio F. Increased tryptase and myeloperoxidase levels in blister fluids of patients with bullous pemphigoid: Correlations with cytokines, adhesion molecules and anti-basement membrane zone antibodies. Exp Dermatol. 2000;9(2):131-7.

9. De Pita O, Frezzolini A, Cianchini G, Ruffelli M, Teofoli P, Puddu P. T-helper 2 involvement in the pathogenesis of bullous pemphigoid: role of soluble CD30 (sCD30). Archives of dermatological research. 1997;289(12):667-70.

10. Dimson OG, Giudice GJ, Fu CL, Van Den Bergh F, Warren SJ, Janson MM, et al. Identification of a potential effector function for IgE autoantibodies in the organ-specific autoimmune disease bullous pemphigoid. J Invest Dermatol. 2003;120(5):784-8.

11. Echigo T, Hasegawa M, Shimada Y, Inaoki M, Takehara K, Sato S. Both Th1 and Th2 chemokines are elevated in sera of patients with autoimmune blistering diseases. Archives of Dermatological Research. 2006;298(1):38-45.

12. Echigo T, Hasegawa M, Inaoki M, Yamazaki M, Sato S, Takehara K. Antiphospholipid antibodies in patients with autoimmune blistering disease. Journal of the American Academy of Dermatology. 2007;57(3):397-400.

13. Fang H, Xue K, Cao T, Li Q, Dang E, Liu Y, et al. CXCL12/CXCR4 Axis Drives the Chemotaxis and Differentiation of B Cells in Bullous Pemphigoid. J Invest Dermatol. 2023;143(2):197-208.e6.

14. Freire PC, Munoz CH, Stingl G. IgE autoreactivity in bullous pemphigoid: eosinophils and mast cells as major targets of pathogenic immune reactants. Br J Dermatol. 2017;177(6):1644-53.

15. Frezzolini A, Cianchini G, Ruffelli M, Cadoni S, Puddu P, De Pita O. Interleukin-16 expression and release in bullous pemphigoid. Clin Exp Immunol. 2004;137(3):595-600.

16. Fujimura T, Kakizaki A, Furudate S, Aiba S. A possible interaction between periostin and CD163+ skin-resident macrophages in pemphigus vulgaris and bullous pemphigoid. Exp Dermatol. 2017;26(12):1193-8.

17. Furukawa F, Kumagai S, Sakamoto Y, Takigawa M, Imamura S. Elevated serum levels of IgE-binding factor/soluble CD23 in bullous pemphigoid. J Dermatol Sci. 1994;7(2):150-4.

18. Giacalone B, D'Auria L, Bonifati C, Ferraro C, Riccardi E, Mussi A, et al. Decreased interleukin-7 and transforming growth factor-beta1 levels in blister fluids as compared to the respective serum levels in patients with bullous pemphigoid: Opposite behavior of TNF-alpha, Interleukin-4 and Interleukin-10. Exp Dermatol. 1998;7(4):157-61.

19. Gunther C, Carballido-Perrig N, Kopp T, Carballido JM, Pfeiffer C. CCL18 is expressed in patients with bullous pemphigoid and parallels disease course. Br J Dermatol. 2009;160(4):747-55.

20. Gunther C, Wozel G, Meurer M, Pfeiffer C. Up-regulation of CCL11 and CCL26 is associated with activated eosinophils in bullous pemphigoid. Clin Exp Immunol. 2011;166(2):145-53.

21. Hatano Y, Katagiri K, Arakawa S, Umeki T, Takayasu S, Fujiwara S. Successful treatment by double-filtration plasmapheresis of a patient with bullous pemphigoid: Effects in vivo on transcripts of several genes for chemokines and cytokines in peripheral blood mononuclear cells. Br J Dermatol. 2003;148(3):573-9.

22. Hu YQ, Zhang JZ. A Comparison for Type 2 Cytokines and Lesional Inflammatory Infiltrations in Bullous Pemphigoid and Atopic Dermatitis. Clin Cosmet Investig Dermatol. 2022;15((Hu, Zhang) Department of Dermatology, Peking University People's Hospital, Beijing, China):2313-21.

23. Inaoki M, Takehara K. Increased serum levels of interleukin (IL)-5, IL-6 and IL-8 in bullous pemphigoid. J Dermatol Sci. 1998;16(2):152-7.

24. Inaoki M, Echigo T, Hayashi H, Nagaoka T, Hasegawa M, Takehara K, et al. Decreased expression levels of CD22 and L-selectin on peripheral blood B lymphocytes from patients with bullous pemphigoid. J Autoimmun. 2006;27(3):196-202.

25. Julio TA, Vernal S, Massaro JD, Silva MC, Donadi EA, Moriguti JC, et al. Biological predictors shared by dementia and bullous pemphigoid patients point out a cross-antigenicity between BP180/BP230 brain and skin isoforms. Immunol Res. 2018;66(5):567-76.

26. Kakinuma T, Wakugawa M, Nakamura K, Hino H, Matsushima K, Tamaki K. High level of thymus and activation-regulated chemokine in blister fluid and sera of patients with bullous pemphigoid. Br J Dermatol. 2003;148(2):203-10.

27. Khil'chenko S, Boch K, van Beek N, Vorobyev A, Zillikens D, Schmidt E, et al. Alterations of Total Serum Immunoglobulin Concentrations in Pemphigus and Pemphigoid: Selected IgG2 Deficiency in Bullous Pemphigoid. Frontiers in Medicine. 2020;7((Khil'chenko, Schmidt, Ludwig) Lubeck Institute of Experimental Dermatology, University of Lubeck, Lubeck, Germany(Khil'chenko, Boch, van Beek, Vorobyev, Zillikens, Schmidt, Ludwig) Center for Research on Inflammation of the Skin, University of Lubeck, Lu):472.

28. Kieffer M, Barnetson St CR. Increased gliadin antibodies in dermatitis herpetiformis and pemphigoid. Br J Dermatol. 1983;108(6):673-8.

29. Kokubu H, Takahashi T, Kabuto M, Kouzaki H, Fujimoto N. Analysis of IL-10 and IL-35 in dipeptidyl peptidase-4 inhibitor-related bullous pemphigoid. Exp Dermatol. 2023((Kokubu, Takahashi, Kabuto, Fujimoto) Department of Dermatology, Shiga University of Medical Science, Otsu, Japan(Kouzaki) Department of Otolaryngology-Head and Neck Surgery, Shiga University of Medical Science, Otsu, Japan).

30. Le Jan S, Plee J, Vallerand D, Dupont A, Delanez E, Durlach A, et al. Innate immune cell-produced IL-17 sustains inflammation in bullous pemphigoid. J Invest Dermatol. 2014;134(12):2908-17.

31. Le Jan S, Muller C, Plee J, Durlach A, Bernard P, Antonicelli F. IL-23/IL-17 axis activates IL-1beta-associated inflammasome in macrophages and generates an auto-inflammatory response in a subgroup of patients with bullous pemphigoid. Front Immunol. 2019;10(AUG):1972.

32. Li Q, Liu Z, Dang E, Jin L, He Z, Yang L, et al. Follicular Helper T Cells (Tfh) and IL-21 Involvement in the Pathogenesis of Bullous Pemphigoid. PLoS ONE. 2013;8(7):e68145.

33. Liu Y, Peng L, Li L, Liu C, Hu X, Xiao S, et al. TWEAK/Fn14 activation contributes to the pathogenesis of bullous pemphigoid. J Immunol. 2017;198(1 Supplement 1).

34. Liu Z, Dang E, Li B, Qiao H, Jin L, Zhang J, et al. Dysfunction of CD19+CD24hiCD27+ B regulatory cells in patients with bullous pemphigoid. Sci Rep. 2018;8(1):703.

35. Maglie R, Mercurio L, Morelli M, Madonna S, Salemme A, Baffa ME, et al. Interleukin-36 cytokines are overexpressed in the skin and sera of patients with bullous pemphigoid. Exp Dermatol. 2023;32(6):915-21.

36. Marzano AV, Tedeschi A, Berti E, Fanoni D, Crosti C, Cugno M. Activation of coagulation in bullous pemphigoid and other eosinophil-related inflammatory skin diseases. Clin Exp Immunol. 2011;165(1):44-50.

37. Messingham KN, Holahan HM, Frydman AS, Fullenkamp C, Srikantha R, Fairley JA. Human eosinophils express the high affinity IgE receptor, FceRI, in bullous pemphigoid. PLoS ONE. 2014;9(9):e107725.

38. Muramatsu K, Zheng M, Yoshimoto N, Ito T, Ujiie I, Iwata H, et al. Regulatory T cell subsets in bullous pemphigoid and dipeptidyl peptidase-4 inhibitor-associated bullous pemphigoid. J Dermatol Sci. 2020;100(1):23-30.

39. Nakashima H, Fujimoto M, Asashima N, Watanabe R, Kuwano Y, Yazawa N, et al. Serum chemokine profile in patients with bullous pemphigoid. Br J Dermatol. 2007;156(3):454-9.

40. Nesmond S, Muller C, Le Naour R, Viguier M, Bernard P, Antonicelli F, et al. Characteristic Pattern of IL-17RA, IL-17RB, and IL-17RC in Monocytes/Macrophages and Mast Cells From Patients With Bullous Pemphigoid. Front Immunol. 2019;10((Nesmond, Muller, Le Naour, Bernard, Antonicelli, Le Jan) Laboratory of Dermatology, EA7509 IRMAIC, University of Reims-Champagne-Ardenne, Reims, France(Le Naour) Department of Biological Sciences, UFR Pharmacy, University of Reims Champagne-Ardenne, Reim):2107.

41. Pruessmann J, Pruessmann W, Holtsche MM, Linnemann B, Hammers CM, Van Beek N, et al. Immunomodulator galectin-9 is increased in blood and skin of patients with bullous pemphigoid. Acta Derm Venereol. 2021;101(3):adv00419.

42. Qian H, Kusuhara M, Li X, Tsuruta D, Tsuchisaka A, Ishii N, et al. B-cell activating factor detected on both naive and memory B cells in bullous pemphigoid. Exp Dermatol. 2014;23(8):596-605.

43. Qiao P, Dang E, Cao T, Fang H, Zhang J, Qiao H, et al. Dysregulation of mCD46 and sCD46 contribute to the pathogenesis of bullous pemphigoid. Sci Rep. 2017;7(1):145.

44. Qiu L, Zhang L, Qi R, Gao X, Chen H, Xiao T. MiR-1291 functions as a potential serum biomarker for bullous pemphigoid. Dis Markers. 2020;2020((Qiu, Zhang, Qi, Gao, Chen, Xiao) Department of Dermatology, First Hospital of China Medical University, National Health Commission Key Laboratory of Immunodermatology, Key Laboratory of Immunodermatology of Ministry of Education, Shenyang, Liaoning, Chin):9505312.

45. Riani M, Le Jan S, Plee J, Durlach A, Le Naour R, Haegeman G, et al. Bullous pemphigoid outcome is associated with CXCL10-induced matrix metalloproteinase 9 secretion from monocytes and neutrophils but not lymphocytes. J Allergy Clin Immunol. 2017;139(3):863-72.e3.

46. Riani M, Muller C, Bour C, Bernard P, Antonicelli F, Le Jan S. Blister fluid induces MMP-9-associated M2-type macrophages in bullous pemphigoid. Front Immunol. 2019;10(AUG):1858.

47. Rifaioglu EN, Sen BB, Ekiz O, Dogramaci AC. Mean platelet volume and eosinophilia relationship in patients with bullous pemphigoid. Platelets. 2014;25(4):264-7.

48. Rudrich U, Gehring M, Papakonstantinou E, Illerhaus A, Engmann J, Kapp A, et al. Eosinophils are a major source of interleukin-31 in bullous pemphigoid. Acta Derm Venereol. 2018;98(8):766-71.

49. Salz M, Haeberle S, Hoffmann J, Enk AH, Hadaschik EN. Elevated IL-31 serum levels in bullous pemphigoid patients correlate with eosinophil numbers and are associated with BP180-IgE. J Dermatol Sci. 2017;87(3):309-11.

50. Schaller J, Schult R, Jeromin T. Increased IgE-levels in bullous pemphigoid correlate to soluble low affinity Fc-II-receptor for IgE and soluble IL-2-receptor. Journal of the European Academy of Dermatology and Venereology. 1995;5(2):163-9.

51. Shen S, Ke Y, Dang E, Fang H, Chang Y, Zhang J, et al. Semaphorin 4D from CD15+ Granulocytes via ADAM10-Induced Cleavage Contributes to Antibody Production in Bullous Pemphigoid. J Invest Dermatol. 2018;138(3):588-97.

52. Shirakata Y, Shiraishi S, Sayama K, Miki Y. Subclass characteristics of IgG autoantibodies in bullous pemphigoid and pemphigus. J Dermatol. 1990;17(11):661-6.

53. Shrikhande M, Hunziker T, Braathen LR, Pichler WJ, Dahinden CA, Yawalkar N. Increased coexpression of eotaxin and interleukin 5 in bullous pemphigoid. Acta Derm Venereol. 2000;80(4):277-80.

54. Suzuki M, Yamaguchi Y, Nakamura K, Kanaoka M, Matsukura S, Takahashi K, et al. Serum thymus and activation-regulated chemokine (TARC/CCL17) may be useful to predict the disease activity in patients with bullous pemphigoid. Journal of the European Academy of Dermatology and Venereology. 2021;35(2):e121-e4.

55. Tedeschi A, Marzano AV, Lorini M, Balice Y, Cugno M. Eosinophil cationic protein levels parallel coagulation activation in the blister fluid of patients with bullous pemphigoid. Journal of the European Academy of Dermatology and Venereology. 2015;29(4):813-7.

56. Teraki Y, Hotta T, Shiohara T. Skin-homing interleukin-4 and -13-producing cells contribute to bullous pemphigoid: Remission of disease is associated with increased frequency of interleukin-10-producing cells. J Invest Dermatol. 2001;117(5):1097-102.

57. Treco E, Huan E, Varzavand A, Fairley JA, Messingham K. Elevated levels of sCD48 are inversely correlated with markers of disease activity in bullous pemphigoid. Exp Dermatol. 2023;32(1):85-90.

58. Watanabe R, Ishiura N, Nakashima H, Yazawa N, Kuwano Y, Tada Y, et al. Increased serum levels of circulating CD40 ligand in patients with bullous pemphigoid: Preliminary results. Dermatology. 2007;215(3):180-6.

59. Watanabe R, Fujimoto M, Yazawa N, Nakashima H, Asashima N, Kuwano Y, et al. Increased serum levels of a proliferation-inducing ligand in patients with bullous pemphigoid. J Dermatol Sci. 2007;46(1):53-60.

60. Zebrowska A, Erkiert-Polguj A, Wagrowska-Danilewicz M, Danilewicz M, Pawliczak R, Sysa-Jedrzejowska A, et al. Eotaxin (CCL11), TARC [thymus and activation-regulated chemokine (CCL17)], MCP-1 (CCL2) and CCR-1, CXCR-1, CXCR-2 expression in dermatitis herpetiformis and bullous pemphigoid. Arch Med Sci. 2009;5(3):475-85.

61. Zebrowska A, Wagrowska-Danilewicz M, Danilewicz M, Stasikowska-Kanicka O, Kulczycka-Siennicka L, Wozniacka A, et al. Mediators of mast cells in bullous pemphigoid and dermatitis herpetiformis. Mediators Inflamm. 2014;2014((Zebrowska, Kulczycka-Siennicka, Wozniacka, Waszczykowska) Department of Dermatology and Venereology, Medical University of Lodz, Plac Hallera 1, Lodz 90-497, Poland(Wagrowska-Danilewicz, Danilewicz, Stasikowska-Kanicka) Laboratory of Nephropathology of M):936545.

62. Zebrowska A, Wagrowska-Danilewicz M, Danilewicz M, Wieczfinska J, Pniewska E, Zebrowski M, et al. Tissue factor in dermatitis herpetiformis and bullous pemphigoid: Link between immune and coagulation system in subepidermal autoimmune bullous diseases. Mediators Inflamm. 2015;2015((Zebrowska, Waszczykowska, Wozniacka) Department of Dermatology and Venereology, Medical University of Lodz, Hallera Square 1, Lodz 90-497, Poland(Wagrowska-Danilewicz, Danilewicz) Laboratory of Nephropathology, Medical University of Lodz, Pomorska 251 St):870428.
