# Supplementary material for: Serum Biomarkers in Bullous Pemphigoid: A Systematic Review
Source: J Cutan Med Surg. 2024 Jul 29;28(5):463–7. doi: 10.1177/12034754241266171 (PMC11514321; doi:10.1177/12034754241266171)
Supplement: sj-docx-2-cms-10.1177_12034754241266171 – Supplemental material for Serum Biomarkers in Bullous Pemphigoid: A Systematic Review [file sj-docx-2-cms-10.1177_12034754241266171.docx]

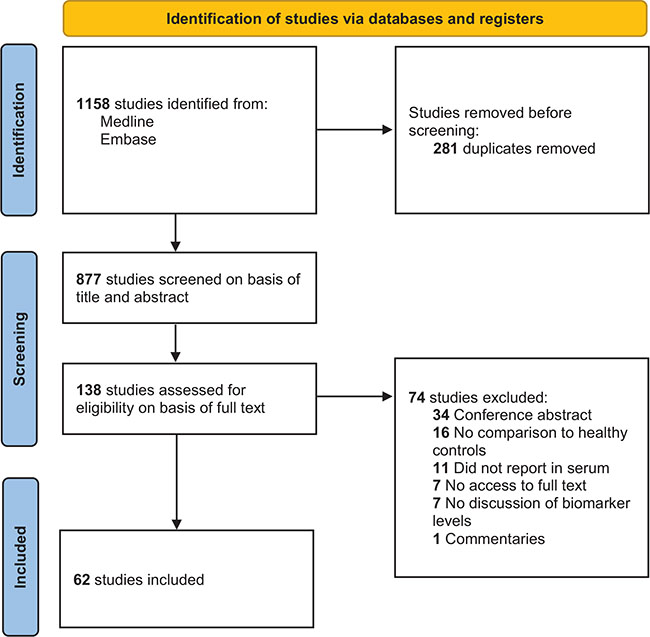


**Figure 1.** Several trigger factors have been identified in BP, including genetic predisposition, UV radiation, drugs, trauma, allergic/hypersensitivity reactions, and infection. Imbalances between T_reg_ and autoreactive T_h_ cell activity can lead to loss of self-tolerance, resulting in production of IgG or IgE autoantibodies against BP180 and BP230. Increased T_h_17 cell activity leads to the activation of neutrophils, eosinophils, and T_h_2 cells, resulting in activation of the coagulation cascade via TF and release of pro-inflammatory cytokines and degradative enzymes. Anti-BP180/230 IgG also activates complement, further stimulating neutrophil and eosinophil activity. Anti-BP180/230 IgE stimulates mast cell activation, contributing to the inflammation, pruritus and blister formation observed in BP. CCL, c-c motif chemokine ligand; ECP, eosinophil cationic protein; F1+2, prothrombin fragment F1+2; IL, interleukin; MMP, matrix metalloprotease; ROS, reactive oxygen species; TF, tissue factor; TLR, toll-like receptor; TGF, transforming growth factor; BP, Bullous pemphigoid.
